# Solitary Fibrous Tumor With an Acute Subdural Hematoma: A Case Report and Review of the Literature

**DOI:** 10.7759/cureus.58271

**Published:** 2024-04-14

**Authors:** Tomoya Ohsaka, Yasuyuki Kojita, Atsushi Urase, Ayumi Hirayama, Minoru Yamada, Sung-Woon IM, Atsushi K Kono, Yasuhiro Sanada, Takaaki Chikugo, Noboru Tanigawa, Kazunari Ishii

**Affiliations:** 1 Department of Radiology, Faculty of Medicine, Kindai University, Osakasayama, JPN; 2 Department of Radiology, Kansai Medical University, Hirakata, JPN; 3 Department of Neurosurgery, Faculty of Medicine, Kindai University, Osakasayama, JPN; 4 Department of Pathology, Faculty of Medicine, Kindai University, Osakasayama, JPN

**Keywords:** hemangiopericytoma (hpc), tumor, brain, subdural hematoma, solitary fibrous tumor (sft)

## Abstract

Solitary fibrous tumor (SFT) is a rare interstitial tumor that originates from various soft tissues, and SFTs occurring within the cranium are extremely rare. While intracranial SFTs with cerebral hemorrhage or subarachnoid hemorrhage have been reported, there have been no reports of intracranial SFTs causing subdural hematoma. In this case, we report on an intracranial SFT accompanied by a subdural hematoma. A 29-year-old female was emergently transported due to the sudden onset of persistent headache and vomiting that began the night before. CT and MRI imaging revealed a hemorrhagic tumor under the tentorium and an acute subdural hematoma extending along the tentorium. The excised tumor was diagnosed as an SFT through histopathological examination. After undergoing radiation therapy, no recurrence has been observed. This is the first case report of an SFT accompanied by a subdural hematoma, and it is vital to recognize that SFTs can be associated with subdural hematomas for proper diagnosis and treatment planning.

## Introduction

Solitary fibrous tumor (SFT) was first reported in 1931 as a tumor originating from the pleura [1] and initially considered a mesenchymal tumor predominantly found in the pleura, but currently, it has been reported in various other sites. SFT originating from the central nervous system was first reported in 1996 [2], and to date, there have been over 200 reported cases [3]. Ninety percent of central nervous system SFTs occur in adults over the age of 30, with 76% developing intracranially and 24% within the spinal canal. The most common intracranial locations for SFTs are the dural convexity at 36%, cerebellar tentorium at 16%, and cerebellopontine angle at 10% [2,3].

Reports of intracranial hemorrhage associated with SFTs are relatively rare, and there have been no reports of cases with acute subdural hematoma. In this report, we present a case of a central nervous system SFT accompanied by a subdural hematoma, providing new insight into the necessity of considering SFT in the differential diagnosis of extracerebral tumors accompanied by subdural hematomas.

## Case presentation

**Figure 1:**
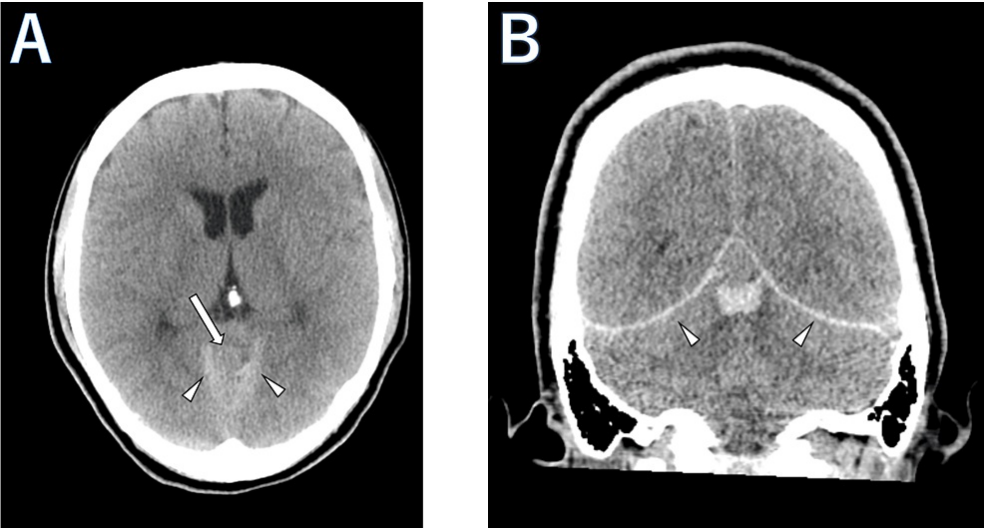
Preoperative non-enhanced computed tomography (CT). (A) Axial and (B) coronal images show a hemorrhagic mass (arrow) measuring 1.6 cm × 1.4 cm × 1.2 cm under the cerebellar tentorium with the isodensity as cortical gray matter, with bleeding observed below the mass. No calcification was present. An acute subdural hematoma extending along the cerebellar tentorium (arrowhead) was demonstrated.

**Figure 2:**
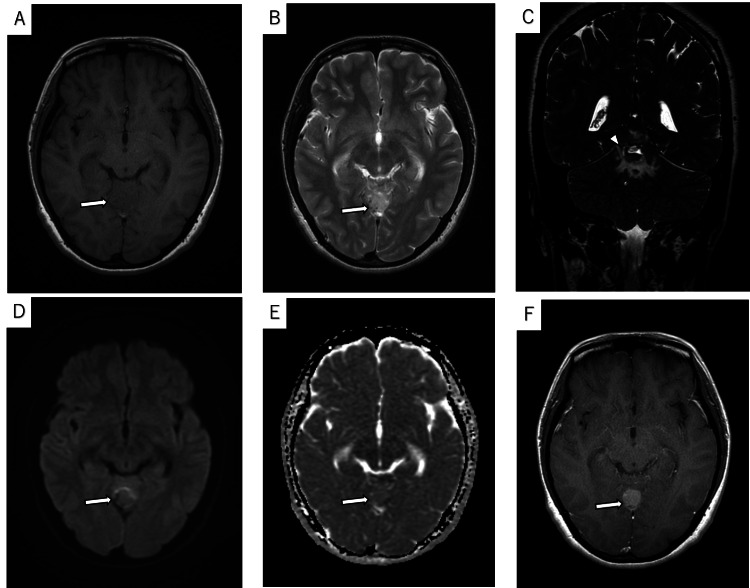
Preoperative magnetic resonance imaging (MRI). (A) Axial T1-weighted image and (B) T2-weighted image demonstrate a tumor (arrow) adjacent to the cerebellar tentorium. The lesion shows isointensity on T1-weighted images and hyperintensity on T2-weighted images compared to cortical gray matter. (C) A 3D T2-weighted turbo spin echo (TSE) image demonstrates the CSF cleft sign (arrowhead). (D) Diffusion-weighted imaging and (E) apparent diffusion coefficient (ADC) indicate slight diffusion restriction of the tumor (arrow), with the minimum ADC value on the ADC map being 0.83 × 10^-3^ mm^2^/s. Normalized ADC (tumor's ADC value/normal cerebral hemispheric white matter ADC value) was 1.13. (F) Contrast-enhanced T1-weighted image shows homogeneous enhancement of the tumor (arrow) without the dural tail sign.

**Figure 3:**
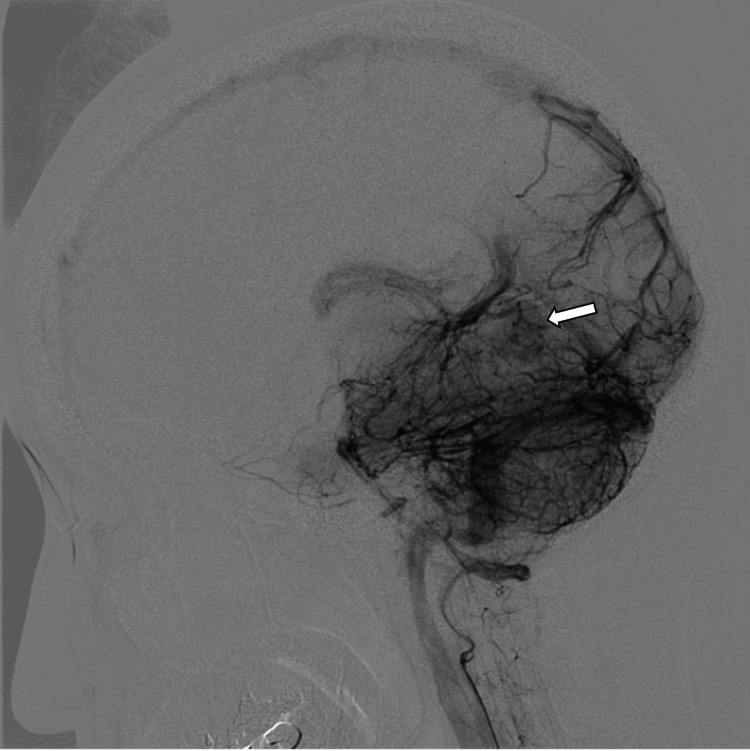
Cerebral digital subtraction angiography (DSA) via a right vertebral artery injection demonstrating a tumor (arrow) located below the straight sinus in the lateral view.

A 29-year-old female was emergently transported due to the sudden onset of persistent headache and vomiting that began the night before. Noncontrast CT revealed a hemorrhagic mass under the tentorium and an acute subdural hematoma extending along the cerebellar tentorium (Figure 1). In MRI, the tumor located under the cerebellar tentorium showed isointensity in the T1-weighted image (T1WI) and hyperintensity in the T2-weighted image (T2WI) compared to cortical gray matter (Figure 2). A CSF cleft sign was observed between the tumor and the cerebellar parenchyma in a 3D T2-weighted turbo spin echo (TSE) image, suggesting the tumor was extracerebral. Diffusion-weighted imaging and apparent diffusion coefficient (ADC) demonstrated slight diffusion restriction, with the normalized ADC (tumor's ADC value/normal cerebral hemispheric white matter ADC value) being 1.13. The tumor exhibited a heterogeneous signal on T1WIs after contrast, with no dural tail sign observed. The tumor was not observed in a noncontrast brain MRI taken two years prior. Cerebral DSA via a right vertebral artery injection reveals a tumor situated below the straight sinus, with no Sunburst appearance observed (Figure 3).

**Figure 4:**
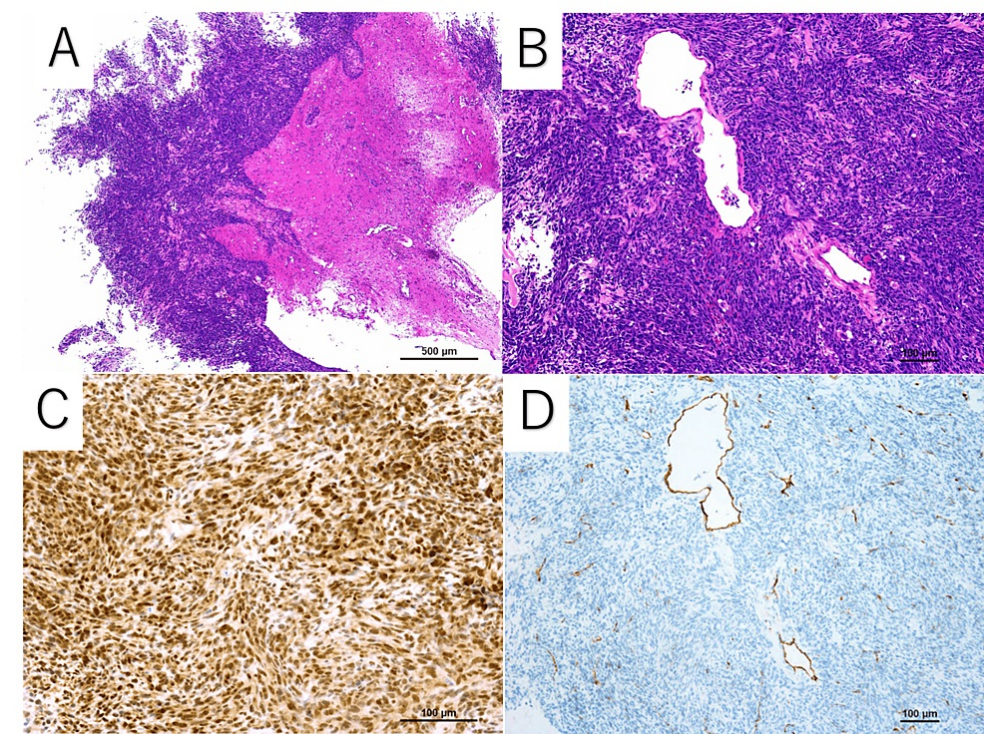
Histological findings. H&E stain (A, low magnification; B, high magnification) showed densely proliferating cells with hyperchromatic spindle to oval nuclei. (C) Immunohistochemical examination revealed strong nuclear expression of STAT6. (D) CD34 was positive only in vessels interspersed among the atypical cells. Ki-67 staining indicated a proliferation index of 55% (not shown).

Subsequently, a craniotomy for tumor resection was performed. The tumor was extradural and adhered to the dura near the straight sinus. Histopathology is shown in Figure 4. H&E staining showed densely proliferating cells with hyperchromatic spindle to oval nuclei, forming large and small nodules with fascicular and loosely whorled patterns. Mitotic activity was also observed. Immunohistochemical examination showed positive staining for STAT6, while EMA and S-100 were negative. CD34 was positive only in the vessels interspersed among the atypical cells. Ki-67 staining showed a proliferation index of 55%. These findings led to the diagnosis of Grade 3 SFT. A follow-up contrast MRI confirmed the removal of the tumor. Symptoms were alleviated after the surgery. As an additional treatment, stereotactic radiotherapy was administered with a total dose of 59.4 Gy, divided into a daily dose of 1.8 Gy over 33 fractions. A temporary onset of mild headaches was experienced during the radiotherapy, which improved within a few days. At the three-month follow-up appointment, the surveillance MRI showed no signs of tumor recurrence.

## Discussion

SFT is a rare tumor that originates from soft tissues throughout the body. In the central nervous system, SFT was historically classified alongside hemangiopericytoma (HPC). However, the 2021 WHO classification of brain tumors has amalgamated these categories, now recognizing them as a single tumor type and designating *SFT* as the exclusive term for this specific pathology [4]. Central nervous system SFTs occur in the spinal canal in 24% of cases and intracranially in 76% of cases. There is no significant gender difference in intracranial SFTs (men, 47%; women, 53%), with the highest incidence in the 51-60 age group [2]. The most common symptoms include headache (50%), followed by gait imbalance, weakness, visual disturbances, cranial nerve deficits, and nausea [2]. In this case, symptoms of headache and vomiting were observed, and the sudden onset suggests that they were related to hemorrhage.

Reports of intracranial hemorrhage, including cerebral and subarachnoid hemorrhage in SFTs, are relatively rare, and our case is the first to report an SFT with acute subdural hematoma. According to previous reports, histopathological studies reveal that 23.4% of SFTs display signs of microscopic intratumoral bleeding [5]. The mechanism of bleeding involves the development of fragile and immature vessels due to tumor growth, changes in the tumor’s vascular distribution known as stag-horn vessels, and endothelial proliferation leading to vessel occlusion and necrosis [6]. In our case, it was unclear whether the subdural hematoma originated from the tumor or surrounding vessels in the subdural space, but the presence of intra-tumoral hemorrhage suggests that the bleeding from the tumor progressed into the subdural space.

On imaging, SFTs are usually well-circumscribed extracerebral tumors. On CT, they show iso- to hyperdensity compared to adjacent brain parenchyma, iso- to hyperintensity on T1WIs compared to the cortical gray matter, and isointensity on T2WIs [7]. A mixed pattern of hyperintensities and hypointensities, appearing as a speckled yin-yang pattern on a T2WI is useful for differentiation; hyperintensities represent areas of high cellularity, and hypointensities represent fibrotic components [8]. In this case, the SFT showed isodensity compared to cortical gray matter on CT, which is a typical finding. On MRI, it exhibited isointensity compared to cortical gray matter on T1WI and hyperintensity on T2WI, with no clear yin-yang pattern observed. Calcification is rare, and cystic changes or necrosis are common [8]. SFTs often have a rich blood supply, showing numerous flow voids, and are usually moderately to highly enhanced [9]. Intracranial SFTs on angiography are supplied by either the external, internal, or both carotid arteries or vertebral-basilar arteries, appearing as moderately to highly neovascularized tumors [3].

Meningiomas, like SFTs, can also cause bleeding, with 45 cases of meningiomas accompanied by subdural hematomas reported so far [10]. Differentiating SFTs from meningiomas in imaging is challenging, and the absence of the dural tail sign, typically seen in meningiomas, suggests SFT [3]. Chen et al. [11] noted that SFTs have significantly higher ADC values than Grades I-III meningiomas, with a normalized ADC threshold >1.15 being useful in differentiation [12,13]. Compared to meningiomas, SFTs tend to have a narrower attachment to the dura, more frequent occurrence of flow voids, and less frequent calcification [7]. In our case, there was no dural tail sign or calcification, the normalized ADC value was below 1.15, and flow voids were not prominent, making preoperative differentiation between meningioma and SFT challenging.

The recurrence rate of intracranial SFTs is higher compared to extracranial SFTs and meningiomas, with nearly a 50% recurrence rate within five years after surgical resection and up to 30% rate of extraneural metastasis postoperatively [12]. When comparing cases of surgery alone to those where radiation therapy (total dose of 44-73.6 Gy delivered in fractions of 1.6-2 Gy) was added postoperatively, it is reported that while there is no difference in overall survival, local control rates improved from 60% to 90% [13]. For cases of incomplete tumor resection, or those with poor prognostic factors (such as atypical histological features like cellular pleomorphism, high cellularity, and high MIB-1 values), biannual MRI follow-up is recommended. Long-term follow-up is advised for all cases, including those with complete resection and without poor prognostic factors [3].

## Conclusions

This report presents the first case of an SFT accompanied by an acute subdural hematoma. While there have been reports of meningiomas associated with subdural hematomas, no such cases have been reported for SFTs until now. Therefore, it is imperative to consider SFTs in the differential diagnosis of extracerebral tumors presenting with acute subdural hematomas in the future. However, to understand how intracranial SFTs may lead to acute subdural hematomas, further reports and studies are necessary.
